# CLEC-2 Prevents Accumulation and Retention of Inflammatory Macrophages During Murine Peritonitis

**DOI:** 10.3389/fimmu.2021.693974

**Published:** 2021-06-07

**Authors:** Joshua H. Bourne, Nonantzin Beristain-Covarrubias, Malou Zuidscherwoude, Joana Campos, Ying Di, Evelyn Garlick, Martina Colicchia, Lauren V. Terry, Steven G. Thomas, Alexander Brill, Jagadeesh Bayry, Steve P. Watson, Julie Rayes

**Affiliations:** ^1^ Institute of Cardiovascular Sciences, College of Medical and Dental Sciences, University of Birmingham, Birmingham, United Kingdom; ^2^ Centre of Membrane Proteins and Receptors (COMPARE), Universities of Birmingham and Nottingham, The Midlands, United Kingdom; ^3^ Institute of Immunology and Immunotherapy, University of Birmingham, Birmingham, United Kingdom; ^4^ Department of Pathophysiology, Sechenov First Moscow State Medical University (Sechenov University), Moscow, Russia; ^5^ Institut National de la Santé et de la Recherche Médicale, Centre de Recherche des Cordeliers, Equipe - Immunopathologie et Immunointervention Thérapeutique, Sorbonne Université, Université de Paris, Paris, France; ^6^ Biological Sciences and Engineering, Indian Institute of Technology Palakkad, Kerala, India

**Keywords:** CLEC-2, podoplanin, macrophage, inflammation, platelet

## Abstract

Platelets play a key role in the development, progression and resolution of the inflammatory response during sterile inflammation and infection, although the mechanism is not well understood. Here we show that platelet CLEC-2 reduces tissue inflammation by regulating inflammatory macrophage activation and trafficking from the inflamed tissues. The immune regulatory function of CLEC-2 depends on the expression of its ligand, podoplanin, upregulated on inflammatory macrophages and is independent of platelet activation and secretion. Mechanistically, platelet CLEC-2 and also recombinant CLEC-2-Fc accelerates actin rearrangement and macrophage migration by increasing the expression of podoplanin and CD44, and their interaction with the ERM proteins. During ongoing inflammation, induced by lipopolysaccharide, treatment with rCLEC-2-Fc induces the rapid emigration of peritoneal inflammatory macrophages to mesenteric lymph nodes, thus reducing the accumulation of inflammatory macrophages in the inflamed peritoneum. This is associated with a significant decrease in pro-inflammatory cytokine, TNF-α and an increase in levels of immunosuppressive, IL-10 in the peritoneum. Increased podoplanin expression and actin remodelling favour macrophage migration towards CCL21, a soluble ligand for podoplanin and chemoattractant secreted by lymph node lymphatic endothelial cells. Macrophage efflux to draining lymph nodes induces T cell priming. In conclusion, we show that platelet CLEC-2 reduces the inflammatory phenotype of macrophages and their accumulation, leading to diminished tissue inflammation. These immunomodulatory functions of CLEC-2 are a novel strategy to reduce tissue inflammation and could be therapeutically exploited through rCLEC-2-Fc, to limit the progression to chronic inflammation.

**Graphical Abstract:**
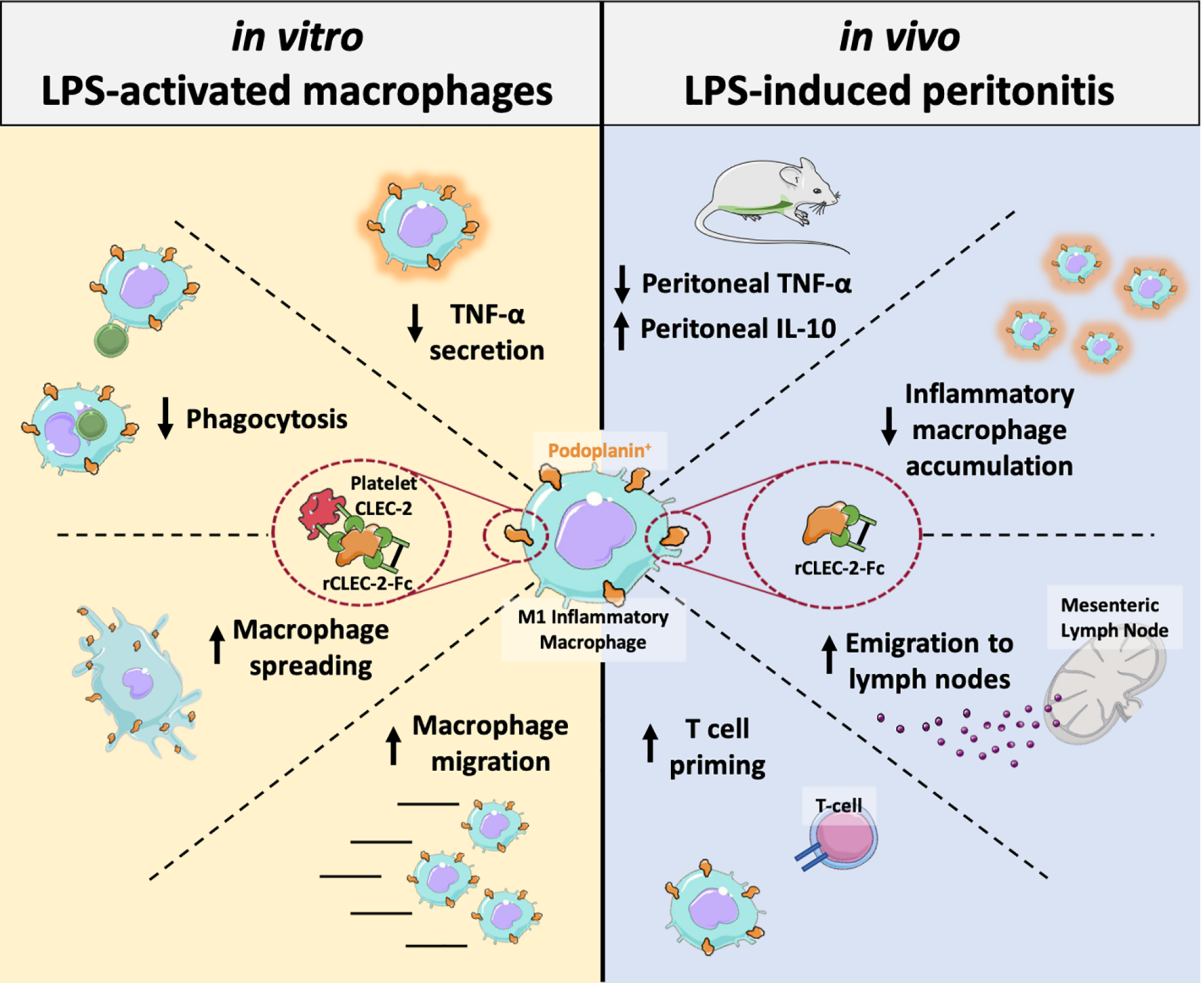


## Introduction

Alongside their role in thrombosis and haemostasis, platelets are emerging as vital regulators of the inflammatory response under sterile and infectious conditions (1, 2). Platelet immunoregulatory functions are tightly regulated by the nature of the insult and the environment. Interestingly, distinct platelet receptors are engaged in different vascular beds and differentially regulate vascular integrity, thrombosis and inflammation (3, 4). Platelet-innate immune cell interactions play a pivotal role in balancing the immune response, however little is known on the functional relevance of these interaction during ongoing inflammation, and how it regulates the resolution of the inflammation. Platelet-leukocyte aggregates are observed in the blood during sterile inflammation and following bacterial, viral and fungal infections (5–8). Platelets are also found in inflamed tissues, such as the lung and peritoneum, primarily in complexes with monocytes, neutrophils and macrophages (3, 9–14). Among the receptors reported to regulate the inflammatory response, the adhesion receptors glycoprotein I (GPIb) and C-type lectin-like receptor 2 (CLEC-2) were shown to differentially regulate macrophage polarization and activation in the inflamed peritoneum, with distinct receptors engaged during the time course of the inflammatory response (10, 12, 13). An increase in macrophage recruitment and activation, drives tissue inflammation observed in atherosclerosis and metabolic disorders, as well as non-resolved infection-driven inflammation (15).

CLEC-2 is a hemi-immunoreceptor tyrosine-based activation motif (hemi-ITAM) receptor constitutively expressed on platelets and a sub-set of dendritic cells. CLEC-2 induces platelet activation through its interaction with its endogenous ligands podoplanin or heme (16, 17). Podoplanin is a small, transmembrane O-glycosylated mucin-type protein constituently expressed on type I lung epithelial cells, fibroblastic reticular cells, lymphatic endothelial cells and podocytes, and is upregulated on inflammatory macrophages, TH17 cells, fibroblasts and cancer cells (18, 19). Beside the role of CLEC-2-podoplanin in thrombosis (20, 21), deletion of platelet-CLEC-2 or haematopoietic-podoplanin increases the cytokine storm and bacterial growth and spreading during caecal ligation and puncture-mediated peritonitis (10, 22). Whether crosslinking podoplanin can regulate macrophage phenotype, fate or resultant tissue inflammation is not known. This is highly relevant in diseases describing platelet-bound podoplanin-positive macrophages such as atherosclerosis (23), rheumatoid arthritis (24), and breast cancer (25).

In this study, we investigate the mechanisms by which CLEC-2 regulates macrophage activation, accumulation and fate during ongoing inflammation. Our study shows a key role for CLEC-2 in macrophage trafficking from the inflamed tissues. This provides a rational to use recombinant CLEC-2-Fc as a therapeutic protein to limit macrophage accumulation in inflamed tissues and progression to chronic inflammation.

## Methods

### Mice

Wild type (WT) C57BL/6 mice (12-14 weeks; males and females) were purchased from Harlan Laboratories (Oxford, UK). Platelet-specific CLEC-2-deficient (26) (CLEC1b^fl/fl^GPIbCre), haematopoietic-specific podoplanin deficient (10) (PDPN^fl/fl^Vav-iCre) and LifeAct-GFP (27) mice were used. All experiments were performed in accordance with UK law (Animal Scientific Procedures Act 1986) with approval of the local ethics committee and UK Home Office approval under PPL P2E63AE7B, PP9677279 and P0E98D513 granted to the University of Birmingham.

### Cell Culture

RAW264.7 cells (Sigma Aldrich) were cultured as previously described (28). Bone marrow cells were isolated from WT mice or PDPN^fl/fl^Vav-iCre^+^ (tibias and femurs) and differentiated into bone marrow-derived macrophages (BMDM) with L-929 conditioned medium for 7 days (29). RAW264.7 cells and BMDM were maintained in Dulbecco’s Modified Eagle Media (DMEM, Thermofisher) supplemented with 10% heat-inactivated foetal bovine serum (FBS), 1% *penicillin*-streptomycin and 2mM L-*glutamine* in a humidified incubator at 5% CO_2_ and 37°C.

### Platelet Preparation

Mouse platelets were prepared as previously described (28).

### Lipopolysaccharide (LPS)-Induced Endotoxemia in Mice

LPS (*Escherichia Coli* 055:B5, Sigma) was injected intraperitoneally (IP) to age- and sex-matched C57BL/6 mice in 200µl saline (10mg/kg). 18h post-LPS-challenge, mice received an intraperitoneal injection of rCLEC-2-Fc or IgG isotype control (100µg/mouse) for an additional 4h. EDTA-treated blood was used to assess blood haematological parameters. Lymph nodes were homogenised, ammonium-Chloride-Potassium (ACK) treated (5mM) and Fc-receptors blocked before antibody staining. Peritoneal cells were collected in 2ml of PBS-EDTA (10mM). Peritoneal lavage fluid (PLF), lymph node cell populations were measured using a CyAn ADP High-Performance Flow Cytometer.

### Immunofluorescent Tissue Staining

Organs were snapped frozen and 6 microns sections used for immunofluorescent staining as previously described (10). Sections were flat-mounted with VECTASHIELD antifade mounting medium (Vector Labs). Antibody mixes are shown in **Supplementary Table 1**. Images were acquired using a Zeiss AxioScan.Z1 microscope and analysed using ZEN software.

## Statistics

All data is presented as mean±SEM. The statistical significance between 2 groups was analyzed using a student’s paired t-test and the statistical difference between multiple groups *in vitro* using one-way ANOVA with Tukey’s multiple comparisons test. The statistical significance for *in vivo* experiments were determined by a Kruskal-Wallis test using Prism 8 (GraphPad Software Inc, USA). Statistical significance was represented by stars: *p < 0.05 **p < 0.01 ***p < 0.001 ****<0.0001.

## Data Sharing Statement

For original data, please contact the corresponding author. Additional methodology available in supplemental methods.

## Results

### Platelet CLEC-2 Delays Inflammatory Macrophage Phagocytic Capacity, Reduces TNF-α Secretion and Accelerates Wound Closure *In Vitro*

Upregulation of podoplanin is observed on inflammatory mouse macrophage cell line, RAW264.7, primary macrophages such as bone marrow-derived macrophages (BMDM), peritoneal macrophage following peritonitis, tumour-associated macrophages and monocyte-derived macrophages in the inflamed lung, liver, skin and other organs (3, 10, 12, 20, 28, 30–32). The expression of podoplanin is associated with macrophage migration. In this study, we assessed the effect of crosslinking podoplanin by platelet CLEC-2 on macrophage function and migration.

**Figure 1:**
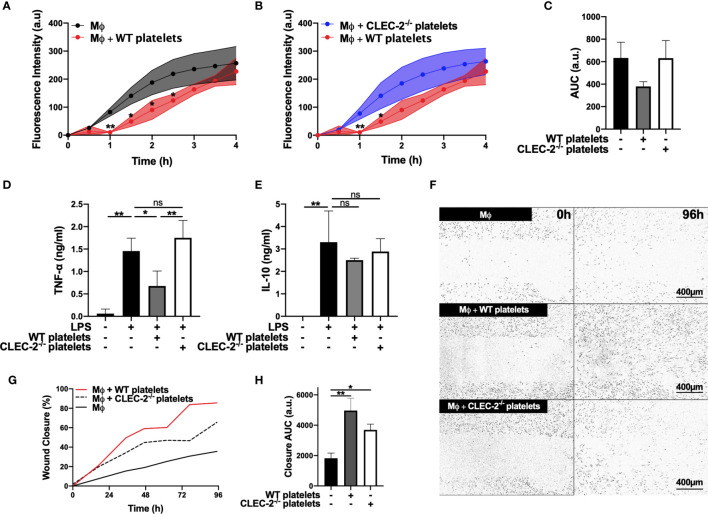
Platelet CLEC-2 delays inflammatory BMDM phagocytic capacity and reduces TNF-α secretion **(A–C)** pH sensitive Alexa Fluor-488 conjugated *Escherichia Coli* bioparticles (3x10^6^ beads/condition) were added to LPS-treated BMDM (Mϕ) in the absence or presence of **(A)** WT platelets or **(B)** CLEC-2-deficient platelets (100 platelets: 1 Mϕ) for 4h. **(A, B)** Phagocytosis was visualised and quantified by time lapse-imaging using an Incucyte Live-cell analysis system. **(C)** Phagocytosis profiles were quantified at 4h by detecting fluorescence/mm^3^ using area under the curve (AUC; a.u. = arbitrary units; n=3). **(D)** TNF-α and **(E)** IL-10 secretion from control Mϕ or in the presence of WT or CLEC-2-deficient platelets was quantified in the supernatant by ELISA (n=4). **(F–H)** Scratch wound migration of Mϕ was monitored every 2h for 96h using an Incucyte ZOOM system. Following wound scratch, **(F)** WT or CLEC-2-deficient platelets (100 platelets:1 Mϕ) were added to Mϕ. **(G)** Wound closure was quantified as percentage of closure using ImageJ. **(H)** Total wound closure was quantified by AUC at 96h (n=3). The statistical significance between 2 groups was analyzed using a student’s paired t-test and the statistical difference between multiple groups using one-way ANOVA with Tukey’s multiple comparisons test. *p < 0.05 **p < 0.01. NS, non-stimulated.

Using BMDM, we show that addition of wild type (WT), but not CLEC-2-deficient platelets (CLEC2^-/-^), to LPS-challenged BMDM delayed the uptake of pH-sensitive fluorescent *E. Coli*-bound bioparticles compared to control, as assessed by Incucyte systems for live-cell microscopy (**Figures 1A–C**). Podoplanin deficiency in BMDM generated from hematopoietic-specific podoplanin-deficient mice was confirmed in the presence on LPS (**Supplementary Figure 1A**). Podoplanin deficiency from BMDM did not affect their phagocytic activity (**Supplemental Figures 1B–C**) suggesting a distinct role for podoplanin-crosslinking by CLEC-2. Crosslinking podoplanin by CLEC-2 expressed on WT platelets inhibited TNF-α secretion from BMDM, whereas the levels of TNF-α were not altered by the addition of CLEC2^-/-^ platelets compared to control (**Figure 1D**). No significant change in IL-10 was observed following platelet addition (**Figure 1E**). In a wound scratch assay, addition of WT platelets to a scratched monolayer of inflammatory BMDM (**Figures 1F–H**) accelerated wound closure by two-fold (36% ±7.4 wound closure for control versus 86% ±12.6 closure in the presence of WT platelets) (**Figures 1F–H**). Wound closure was also accelerated in the presence of CLEC2^-/-^ platelets, although the maximal coverage did not exceed 66% ±18.2. Podoplanin deficiency did not significantly alter wound closure, compared to WT BMDM (**Supplementary Figures 1D–F**), nor did the addition of WT or CLEC-2^-/-^ platelets (**Supplementary Figures 1D, G–H**).

Together these data suggest that platelet CLEC-2 binding to inflammatory BMDM decreased their inflammatory phenotype and regulated their migration.

### Platelet CLEC-2 Upregulates the Expression of Inflammatory Macrophage-Podoplanin to Drive Actin Remodelling

**Figure 2:**
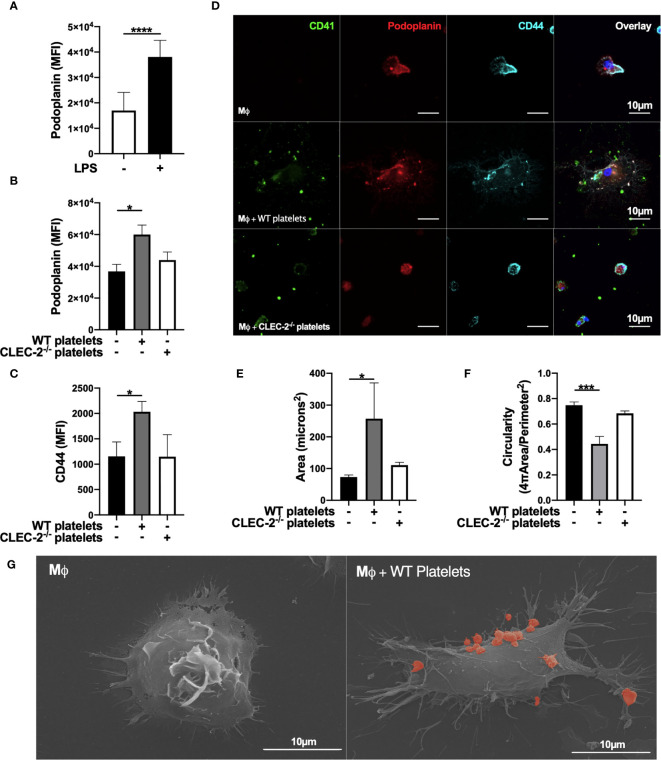
Platelet CLEC-2 upregulates podoplanin and CD44 expression on LPS-stimulated BMDM. **(A)** BMDM were incubated in the presence or absence of LPS (1µg/ml) for 24h. **(B, C)** LPS-stimulated BMDM (Mϕ) were co-cultured in the absence or presence of WT or CLEC-2-deficient platelets (100 platelets: 1 Mϕ). **(A–C)** Median of fluorescence intensity (MFI) of podoplanin (n=4) and CD44 (n=3) was detected by flow cytometry. **(D)** Mϕ were cultured on glass, and WT or CLEC-2-deficient platelets were added for 1h. Platelets (CD41, green), podoplanin (red) and CD44 (cyan) were detected using confocal microscopy. Images are representative of 4 independent experiments. **(E)** Cell area and **(F)** circularity were analysed using ImageJ. **(G)** Mϕ were cultured on glass in the presence or absence of WT platelets (red) for 1h, before fixing and imaging by electron microscopy. The statistical significance between 2 groups was analyzed using a student’s paired t-test and the statistical difference between multiple groups using one-way ANOVA with Tukey’s multiple comparisons test. *p < 0.05, ***p < 0.001, ****p < 0.0001.

We assessed the effect of CLEC-2 on the expression and distribution of podoplanin, and podoplanin transmembrane and intracellular ligands CD44 and ezrin-radixin-moesin (ERM) proteins, respectively. Addition of WT, but not CLEC2^-/-^ platelets for 1h to LPS-stimulated BMDM potentiates podoplanin and CD44 expression, as assessed by flow cytometry (**Figures 2A–C**). The expression of classical activation markers, such as CD80 and CD86, were not altered (not shown). The distribution of podoplanin and CD44 was also altered by CLEC-2, associated with increased BMDM spreading, measured by increased cell area and loss of circularity (**Figures 2D–F**). Platelet-mediated cell spreading and pseudopods formation was confirmed using scanning electron microscopy (**Figure 2G**). Podoplanin and CD44 colocalized on the pseudopod of BMDM in the presence of WT but not CLEC2^-/-^ platelets. Similar effects on podoplanin expression, distribution and spreading was observed in RAW264.7 cells (**Supplementary Figures 2A–C**). The podoplanin intracellular tail contains 2 serines, 167 and 171, that are constitutively phosphorylated and are critical for the association with the ERM proteins and cell migration (33, 34). Immunoprecipitation of podoplanin demonstrated a reduction in the presence of podoplanin phospho-serine residues in the presence of WT but not CLEC2^-/-^ platelets, which promotes podoplanin association with the ERM proteins (**Supplementary Figure 2D**).

In order to confirm the role of platelet on actin remodelling, we imaged LifeAct-GFP-derived inflammatory BMDM in the presence of platelets using diSPIM LightSheet microscopy for 1h. As expected, inflammatory macrophages are sessile, and addition of WT platelets to LPS-activated BMDM increased pseudopod formation, actin remodelling and mobility, compared to control (**Supplementary Video 1, 2**). In contrast, actin remodelling, spreading and pseudopod formation decreased after the phagocytosis of platelets, showing distinct mechanism of cell-cell interaction and platelet phagocytosis on actin remodelling.

These results show that platelet CLEC-2 binding to podoplanin on inflammatory BMDM, or RAW264.7 cells, increases the expression and distribution of podoplanin and its transmembrane ligand CD44, promoting actin remodelling and cell mobility.

### CLEC-2-Mediated Platelet Activation and Secretion Are Dispensable for the Immunomodulatory Functions of CLEC-2

**Figure 3:**
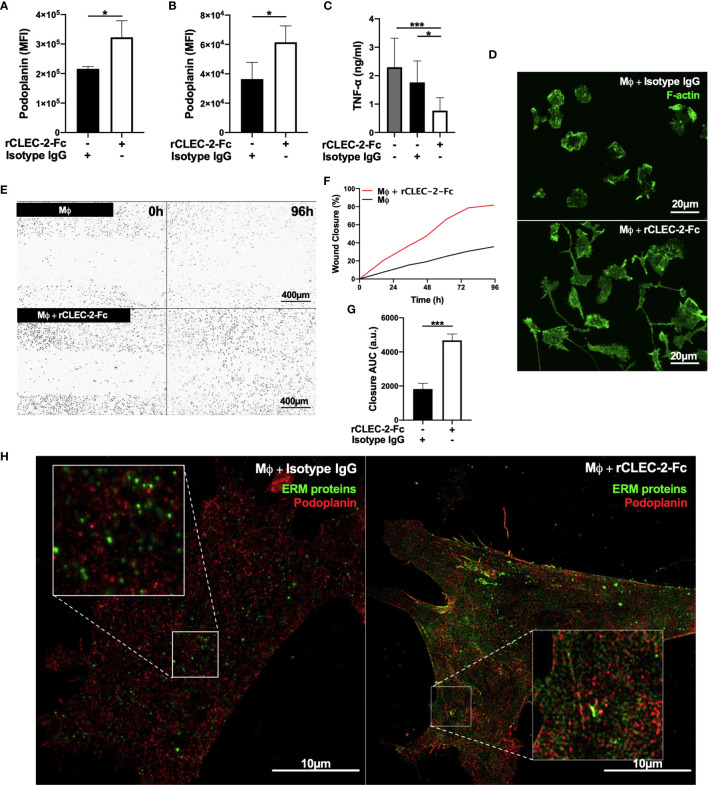
rCLEC-2-Fc upregulates podoplanin expression, and promotes macrophage spreading and migration *in vitro.* **(A, B)** Surface podoplanin expression was detected by flow cytometry using a conjugated anti-podoplanin antibody. 24h LPS-stimulated (1µg/ml) **(A)** RAW264.7 cells (n=3) or **(B)** BMDM (Mϕ; n=4), were washed and incubated with recombinant CLEC-2-Fc (rCLEC-2-Fc; 10µg/ml) or IgG isotype control (10µg/ml) for 1h. **(C)** Mϕ were washed, and cultured with rCLEC-2-Fc (10µg/ml) or IgG isotype control (10µg/ml) for 2h before TNF-α secretion was quantified in the media supernatant by ELISA (n=4). **(D)** LifeAct-GFP-derived Mϕ were spread on collagen in the presence of rCLEC-2-Fc or IgG isotype control (10µg/ml) for 2h and measured with immunofluorescence by confocal microscopy. **(E–G)** Scratch wound migration of Mϕ was monitored every 2h for a total of 96h using an Incucyte ZOOM system. Following wound scratch, **(E)** rCLEC-2-Fc (10µg/ml) was added to Mϕ. **(F)** Wound closure was quantified as percentage of closure compared to initial scratch size using ImageJ. **(G)** Total wound closure was quantified using area under the curve (AUC) at 96h (a.u.= arbitrary units; n=3). **(H)** Mϕ were cultured on collagen in the presence of rCLEC-2-Fc or IgG isotype control (10µg/ml) for 2h. Podoplanin (red) and ERM protein (green) localisation was visualised using immunofluorescence by 3D-structured illumination microscopy (3D-SIM). The statistical significance between 2 groups was analyzed using a student’s paired t-test and the statistical difference between multiple groups using one-way ANOVA with Tukey’s multiple comparisons test. *p < 0.05 ***p < 0.001.

In order to assess whether CLEC-2-dependent platelet activation and secretion, or crosslinking podoplanin is responsible for the immunomodulatory functions of CLEC-2, we performed similar experiments using recombinant dimeric CLEC-2 (rCLEC-2-Fc) and IgG control (17). Addition of rCLEC-2-Fc to LPS-stimulated RAW264.7 cells or LPS-treated BMDM for 1h increased podoplanin expression similar to WT platelets (**Figures 3A, B**). Crosslinking podoplanin with rCLEC-2-Fc decreased TNF-α secretion (**Figure 3C**). In line with this, rCLEC-2-Fc reduced the levels of iNOS, a marker of M1 inflammatory macrophages, without altering Early Growth Response Gene-2 (Egr-2), a marker of M2 anti-inflammatory macrophages (**Supplementary Figures 3A, B**), suggesting a decrease in BMDM inflammatory phenotype without altering their polarization.

rCLEC-2-Fc induced LPS-stimulated BMDM elongation along collagen fibres compared isotype IgG control (**Figure 3D**) and accelerated wound closure (**Figures 3E–G**), confirming the role of podoplanin crosslinking on macrophage migration. The addition of rCLEC-2-Fc to podoplanin-deficient BMDM did not induce wound closure (**Supplementary Figures 4A–C**). Podoplanin was shown to bind to ERM proteins to regulate cell migration. We therefore investigated the interaction and distribution of the ERM proteins and podoplanin in inflammatory macrophages using 3D-SIM microscopy. Surprisingly, podoplanin did not colocalize with the ERM proteins in LPS-treated BMDM. However, addition of rCLEC-2-Fc increased the expression of the ERM proteins and their colocalization with podoplanin (**Figure 3H**), with an enrichment on the filopodia and migrating edges of the cell.

Altogether, our results show that crosslinking podoplanin by CLEC-2, rather than platelet activation and secretion, is responsible for the immunomodulatory effect of CLEC-2 regulating macrophage inflammatory phenotype and their migration.

### rCLEC-2-Fc Decreases Inflammatory Macrophage Accumulation in the Peritoneum During Ongoing Inflammation Induced by LPS

The accumulation of podoplanin-positive macrophages is observed in many infectious and inflammatory diseases such as peritonitis, lung inflammation, liver infection and atherosclerosis (10, 12, 20, 23, 30). We assessed the relevance of crosslinking podoplanin using rCLEC-2-Fc during ongoing peritonitis induced by LPS on the accumulation of inflammatory macrophages in the inflamed peritoneum. WT mice were intraperitoneally injected with LPS or saline for 18h to allow inflammatory macrophage accumulation in the inflamed peritoneum, and rCLEC-2-Fc or IgG isotype control were injected for an additional 4h.

**Figure 4:**
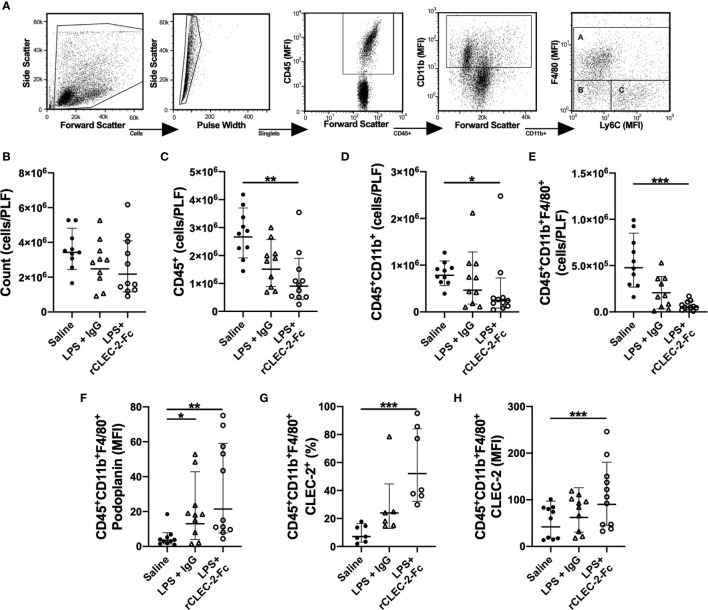
rCLEC-2-Fc decreases macrophage number in the peritoneum following endotoxemia. WT mice were intraperitoneally injected with LPS (10mg/kg) for 18h followed by rCLEC-2-Fc or IgG isotype control (100µg/mouse) for and additional 4h (n=11). Immune cell and platelet infiltration in the peritoneal lavage (PLF) were measured using flow cytometry. **(A)** Gating strategy to identify A: macrophages (CD11b^+^F4/80^+^), B: monocytes (CD11b^+^Ly6C^+^) and C: neutrophils (CD11b^+^F4/80^-^Ly6C^-^). **(B)** Total number of cells from the peritoneal lavage, **(C)** leukocytes (CD45^+^), **(D)** myeloid cells (CD45^+^CD11b^+^) and **(E)** macrophages (CD45^+^CD11b^+^F4/80^+^) were measured in the PLF. **(F)** MFI of podoplanin expressed on the surface of macrophages. **(G)** Percentage of CLEC-2-positive macrophages and **(H)** the MFI of CLEC-2 expression on macrophages. The statistical significance was analyzed using a Kruskal-Wallis multiple comparisons test. *p < 0.05, **p < 0.01, ***p < 0.001.

Myeloid and lymphoid immune cell populations were detected in the peritoneal lavage using flow cytometry. Gating strategy to identify F4/80+ macrophages is shown in **Figure 4A**. At 22h post LPS, no significant change in the total number of cells in the peritoneum was observed in LPS-treated mice compared to control mice (**Figure 4B**). LPS injection did not significantly alter CD45^+^ cell count in the PLF compared to saline-treated mice. However, a significant reduction in CD45^+^ was observed in the peritonitis group treated with rCLEC-2-Fc (**Figure 4C**), more specifically CD11b^+^ F4/80^+^ (**Figures 4D, E**). The reduction in inflammatory F4/80^+^ cells was not due to increased cell apoptosis or death, measured by AnnexinV and Sytox staining (**Supplementary Figure 5A**). Podoplanin expression on F4/80^+^ cells detected in the peritoneum was increased in the LPS groups compared to control, with no significant changes between rCLEC-2-Fc and IgG treatment (**Figure 4F**). LPS increased platelet-macrophage complexes in the PLF, but this was not altered by rCLEC-2-Fc (**Supplementary Figure 5B**). However, a significant increase in F4/80^+^CLEC-2^+^ macrophages was observed following rCLEC-2-Fc treatment, confirming the binding of rCLEC-2-Fc to podoplanin on macrophages, without alteration in platelet-macrophage interactions (**Figures 4G, H**). rCLEC-2-Fc did not alter neutrophil, monocyte, T cells (CD4^+^ and CD8^+^), CD19^+^ or CD45^+^CD11b^-^CD11c^+^ in the PLF (**Supplementary Figures 5C–H**), suggesting a preferential effect of rCLEC-2-Fc on inflammatory F4/80^+^ cells. rCLEC-2 treatment did not induce bleeding in the peritoneum as measured using red blood cell marker Ter 119 (**Supplementary Figure 5I**).

These results show that rCLEC-2-Fc preferentially alters peritoneal podoplanin-positive inflammatory macrophage accumulation and retention in the inflamed peritoneum.

### rCLEC-2-Fc Treatment Reduces TNF-α and Increases IL-10, CCL2, CCL5 and CXCL1 Levels in the Inflamed Peritoneum

We assessed whether the change in macrophage accumulation in the inflamed peritoneum is accompanied by an alteration in cytokine and chemokine release. Similar to our *in vitro* observations, rCLEC-2-Fc decreased TNF-α secretion in the PLF of LPS-treated mice (**Supplemental Figure 6A**) and increased the levels of the anti-inflammatory cytokine IL-10 (**Supplementary Figure 6B**) and the chemokines CCL2, CCL5 and CXCL1 (**Supplementary Figures 6C–E**). The level of CCL21, the soluble ligand for podoplanin, was not significantly altered in the peritoneum in rCLEC-2-Fc-treated mice (**Supplemental Figure 6F**). The emigration of macrophages was not associated with increased vascular permeability, as measured by angiopoietin-2 secretion (**Supplementary Figure 6G**). There was no change in MMP-9, CXCL2, CCL4, C5a, IL-6 or IL1-β secretion following rCLEC-2-Fc treatment (**Supplementary Figures 6H–L**).

Together, our results show that reduction in macrophage accumulation is associated with an alteration in the inflammatory environment, in particular a reduction in TNF-α levels and increase in IL-10.

### rCLEC-2-Fc Promotes Peritoneal Macrophage Emigration to Mesenteric Lymph Nodes

**Figure 5:**
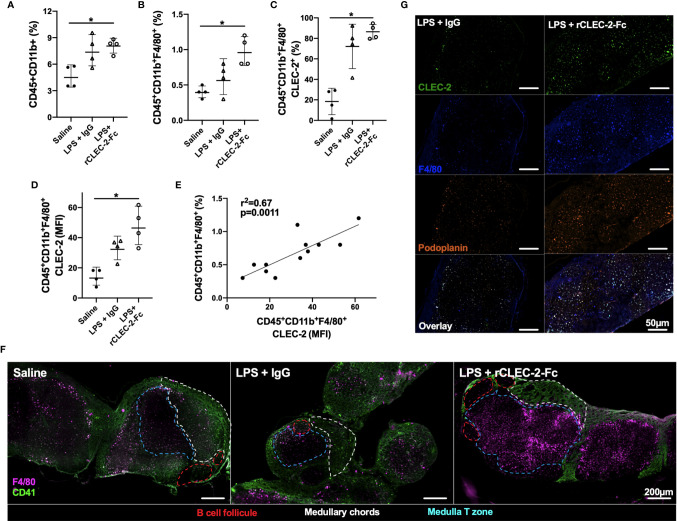
rCLEC-2-Fc increases peritoneal macrophage migration to mesenteric lymph nodes during endotoxemia. WT mice were intraperitoneally injected with LPS (10mg/kg) for 18h followed by rCLEC-2-Fc or IgG isotype control (100µg/mouse) for an additional 4h (n=4). **(A–E)** Mesenteric lymph node (MLN) cells were collected and immune cell population detected by flow cytometry. **(A)** Percentage of myeloid cells (CD45^+^CD11b^+^) and **(B)** macrophages (CD45^+^CD11b^+^F4/80^+^). **(C)** CLEC-2-positive macrophages and **(D)** the MFI of CLEC-2 expression on macrophages in MLN. **(E)** Correlation between the percentage of MLN macrophages and the MFI of CLEC-2 expression on macrophages by Simple Linear Regression. **(F)** Immunofluorescent staining of macrophages (F4/80; purple) and platelets (CD41; green) in frozen MLN sections. **(G)** Immunofluorescent staining of macrophages (F4/80; blue), CLEC-2 (green) and podoplanin (orange) in frozen MLN sections. The statistical significance was analyzed using a Kruskal-Wallis multiple comparisons test. *p < 0.05.

We next investigated the infiltration of inflammatory peritoneal macrophages to the draining lymph nodes. F4/80^+^ population was detected in the mesenteric lymph nodes (MLN) by flow cytometry and immunofluorescence. A significant increase in myeloid cells, in particular F4/80^+^CLEC2^+^, was observed in rCLEC-2-Fc treated mice compared to IgG control (**Figures 5A–D**). The increase in CLEC-2 MFI might be due to the enhanced podoplanin expression, increasing the binding sites for CLEC-2 (**Figure 5D**). The increase in CLEC-2 density on F4/80^+^ cells positively correlated with F4/80^+^ frequency in the MLN (r^2 =^ 0.67, p=0.0011; **Figure 5E**).

These results show that rCLEC-2-Fc binding to inflammatory peritoneal macrophages promotes their emigration from the inflamed peritoneum to the MLN.

### rCLEC-2-Fc-Driven Macrophage Migration to Mesenteric Lymph Nodes Prime T Cells

In order to assess whether emigrated macrophages prime T cells in the draining lymph nodes, we first assessed the location of these macrophages. MLNs were collected 22h post-LPS challenge and macrophages (F4/80^+^) and platelets (CD41^+^) localisation in the lymph nodes was assessed using immunofluorescence (**Figure 5F**). In unchallenged lymph nodes, platelets were localised in the medulla and around the medullary chords. Following LPS challenge, there was no significant staining for F4/80^+^ cells. However, following rCLEC-2-Fc treatment, a significant influx of F4/80^+^ cells was observed in the draining lymph nodes with a concentration of cells in the medulla, in close contact with T cells. We did not detect macrophages bound to platelets in this zone, however macrophages localised in the lymph nodes post-rCLEC-2-Fc are seen to be CLEC-2- and podoplanin-positive compared to IgG control (**Figure 5G**).

**Figure 6:**
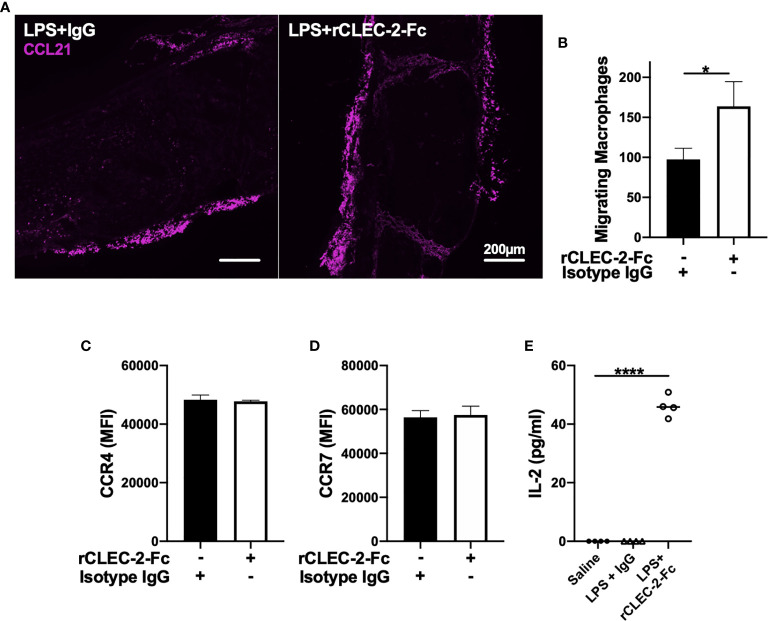
rCLEC-2-Fc increases inflammatory macrophage migration towards CCL21 and primes T cells in mesenteric lymph nodes. WT mice were intraperitoneally injected with LPS (10mg/kg) for 18h followed by rCLEC-2-Fc or IgG isotype control (100µg/mouse) for an additional 4h (n=4). **(A)** Immunofluorescent staining of CCL21 (purple) in frozen MLN taken from LPS-challenged mice. **(B, C)** 24h LPS-stimulated (1µg/ml) BMDM were treated with rCLEC-2-Fc (10µg/ml) or IgG isotype control (10µg/ml) for 4h. Surface expression of **(B)** CCR4 and **(C)** CCR7 median fluorescence intensity (MFI) was detected by flow cytometry (n=4). **(D)** Migration of inflammatory BMDM co-cultured with rCLEC-2-Fc or IgG Isotype control (10µg/ml) toward CCL21 (30ng/ml) was analysed using a Boyden chamber assay for 4h (n=3). **(E)** IL-2 secretion from MLN cells isolated from different mice cultured *in vitro* with LPS (10ng/ml) for 16h was quantified by ELISA.The statistical significance was analyzed using a Kruskal-Wallis multiple comparisons test. *p < 0.05, ****p < 0.0001.

We next investigated whether increased macrophage influx in the MLN is due to an increase in CCL21, a chemoattractant secreted from lymphatic endothelial cells (LECs) and soluble ligand for podoplanin (35). However, we did not observe alteration in CCL21 expression in MLN was observed upon rCLEC-2-Fc treatment (**Figure 6A**). In order to assess whether increased podoplanin expression and actin remodelling increases macrophage chemoattraction towards CCL21, we evaluated LPS-treated macrophage migration towards CCL21 *in vitro* using a Boyden chamber. Addition of rCLEC-2-Fc to LPS-treated BMDM increased the number of macrophages migrating towards CCL21 (**Figure 6B**). This was not due to an increase in CCL21 receptors CCR4 and CCR7 (**Figures 6C, D**). This suggested that CLEC-2-dependent podoplanin and CD44 upregulation, associated with accelerated actin rearrangement, was responsible for increased migration towards CCL21.

In order to assess whether macrophage infiltration into lymph node medulla primes T cells, we stimulated homogenised lymph nodes from different groups *ex vivo* with low-dose LPS (10ng/ml) for 24h. IL-2 secretion, as a readout of T cell priming, was measured in the supernatants by ELISA (**Figure 6E**). Low-dose LPS did not induce IL-2 secretion in MLN cells isolated from saline- or LPS-treated mice. In contrast, a significant increase in IL-2 levels was observed in rCLEC-2-Fc treated mice, suggesting that increased macrophage efflux to the medulla T zone promotes T cell priming.

Altogether, these results suggest that rCLEC-2-Fc alters the expression of podoplanin and its interaction with ligands CD44, ERM and CCL21. This accelerates the removal of inflammatory macrophages from the inflamed peritoneum and their emigration to mesenteric lymph nodes to promote T cell priming, reducing peritoneal inflammation. 

## Discussion

In this study, we report that crosslinking podoplanin using recombinant CLEC-2-Fc limits the inflammatory environment by reducing inflammatory macrophage accumulation in the inflamed tissue and reduces their inflammatory phenotype. During ongoing peritonitis induced by LPS, rCLEC-2-Fc accelerates the removal of inflammatory macrophages from the site of inflammation to the draining lymph nodes and induces T cell priming. The interaction of CLEC-2 with podoplanin upregulated on inflammatory macrophages promotes their mobility through (i) the dephosphorylation of podoplanin intracellular serine residues, (ii) upregulation and rearrangement of podoplanin and CD44 expression on macrophage membrane protrusions, (iii) reorganisation of actin cytoskeleton and ERM protein distribution on cell protrusions and (iv) inflammatory macrophage interaction with CCL21 secreted by lymphatic endothelial cells (LECs). In parallel, CLEC-2 crosslinks podoplanin and reduces TNF-α secretion from inflammatory macrophages and delays their phagocytic activity without changing macrophage polarisation, suggesting a reduction in their inflammatory phenotype. We propose that during acute, ongoing inflammation, CLEC-2-podoplanin crosslinking reduces tissue inflammation by reducing accumulation and retention of inflammatory macrophages and promotes their emigration to draining lymph nodes.

During inflammation, macrophages play three main functions: phagocytosis of debris and dead/apoptotic cells and pathogens, antigen presentation and immunoregulation by secreting an arsenal of cytokines and chemokines. After the first inflammatory phase subsides, macrophages also play a role in tissue repair and wound healing (36). Alteration in the acute or repair phases can lead to chronic inflammation and pathogenic fibrosis. Recent studies showed that platelet interaction with macrophages can alter macrophage function, dependant on the receptor, insult, organ and disease progression (13, 14, 37–40). Using acute inflammatory models, we and others have shown a significant upregulation of podoplanin on inflammatory macrophages (10, 12, 28, 30, 31). Here we show that podoplanin expressed on inflammatory macrophages can be targeted to regulate local inflammation and macrophage trafficking from the site of inflammation. Although many studies have shown that platelet secretion is the main regulator of macrophage function, partly through Prostaglandin E_2_ (11, 40), we show for the first time that the immunomodulatory effect of CLEC-2 is not dependent on platelet activation and secretion, but rather through crosslinking podoplanin. Deletion of CLEC-2 from platelets did not alter the binding of platelets to inflammatory macrophages, suggesting that CLEC-2 is not involved this heterotypic interaction, but exerts an immunomodulatory effect. GPIb (13), CD40L (41), P-selectin (42) or other receptors might be responsible for binding and differentially regulate macrophage functions.

Our study describes a novel mechanism by which crosslinking podoplanin with CLEC-2 reduces tissue inflammation. The use of rCLEC-2-Fc overcomes the limitation of using platelets during ongoing inflammation, in particular due the differential roles of platelet receptors and secretory repertoire in inflammation which can limit clinical translation. Platelet CLEC-2, as well as rCLEC-2-Fc, induces a rapid translocation of podoplanin from intracellular stores to the surface of inflammatory macrophages. This is associated with a loss of phosphorylation of the serine residues in the intracellular podoplanin tail, which has been demonstrated to promote fibroblast migratory activity (33, 34). Classically, macrophages migrate through actin polymerisation-driven elongation of the leading edge towards a gradient, followed by integrin mediated adhesion to matrix proteins and finally actomyosin contraction and trailing edge de-adhesion (43). CLEC-2 induced the reorganisation of the actin cytoskeleton and increased podoplanin interaction with ERM proteins and CD44, promoting macrophage migration. Indeed, CD44 expression is required for podoplanin-induced migration in squamous stratified epithelia (44), suggesting that CLEC-2 may increase podoplanin and CD44 expression and their association, leading to macrophage migration. This cell-specific strategy to limit the accumulation of highly inflamed macrophages in tissues does not induce inflammatory bleeding or thrombosis, which are the major complications associated with platelet-targeting.

Peritoneal macrophages are comprised of 2 functionally and phenotypically distinct subsets, the large and small peritoneal macrophages (LPM and SPM). LPM (F4/80^high^CD11b^high^Ly6C^-^) largely outnumber SPM (F4/80^low^CD11b^low^Ly6C^+^), and are responsible for phagocytosis of cell debris and apoptotic cells and mediate tissue repair (45). In a model of liver sterile injury, in a recent study showed that a sub-population of resident peritoneal macrophages, GATA-6^+^, is rapidly mobilised to the injured liver in CD44-dependent manner, expressing M2 macrophage markers and promoting tissue repair (46). Following intraperitoneal inflammation, LPM rapidly migrate to draining lymph nodes, whereas SPM numbers increases with monocyte influx from the circulation into the peritoneum. These monocytes clear apoptotic neutrophils and subsequently differentiate into macrophages or dendritic cells. Monocyte-derived inflammatory macrophages upregulate podoplanin in response to inflammatory stimuli such as Zymosan and LPS. During the resolution of inflammation, monocytes infiltration and macrophage removal regulates peritoneal inflammation. Macrophage removal could be through local death and/or increased migration to draining lymph nodes (47–49). Here we show that the expression of podoplanin on peritoneal inflammatory macrophages can be targeted to accelerate their removal from the peritoneum. Crosslinking podoplanin using recombinant CLEC-2 induces a series of intracellular changes and receptor redistribution on the cell membrane, increasing their migration. The absence of macrophages from the inflamed peritoneum is not secondary to (i) macrophage local death (49), (ii) increased macrophage adherence through integrin α_D_β_2_ and α_M_β_2_ (Mac-1) upregulation (50) but rather due to macrophage emigration to a secondary site (48), in particular draining lymph nodes. The increase in macrophage emigration was not due to matrix metalloprotease 9 secretion (51), or dysregulated vascular integrity, neither by increase in other chemotactic molecules such as complement C5a levels (22). Combining our *in vitro* and *in vivo* data suggests that a chemoattractant released from the draining lymph nodes is responsible for the directed migration. The directed migration to the draining lymph nodes suggests a potential role for CCL21, constituently secreted by LECs (35) and soluble ligand for podoplanin. CCL21 has previously been described as a key regulator of podoplanin^+^ dendritic cells migrating to lymph nodes (52, 53). This directed migration and retention in lymph nodes represents advantages, including limitation of local peritoneal and potential development of an adaptive immune response.

rCLEC-2-mediated macrophage migration to draining lymph nodes was not restricted to reduce peritoneal inflammation and macrophage accumulation, but also increased T cell priming in the lymph nodes. Macrophages are not solely innate immune cells but can also process and present antigens to naïve T cells in secondary lymphoid organs, driving CD4^+^ T helper cell activation and polarisation to Th1, Th2 or Th17 effector cells. The co-localisation of macrophages with T cells in the medulla increases antigen presentation and T cell priming, observed by increased IL-2 secretion from MLN cells isolated from rCLEC-2-Fc-treated mice. These results suggest that rCLEC-2 not only limits the accumulation of inflammatory macrophages in the peritoneum, but can also direct macrophages to the draining lymph nodes and prime T cell activation. Whether macrophages function as antigen presenting cells, express co-stimulatory molecules or release activating cytokines to prime T cells needs further investigation. Moreover, T cell priming, polarisation and survival following clonal expansion requires further investigation. The beneficial targeting of macrophage emigration to lymph nodes is dependent on the disease, as the association of podoplanin^+^ macrophages with tumour lymphatic vessels in mammary tumour model correlates with increased lymph node and distant organ metastasis (32).

We have previously shown that platelet-CLEC-2-deficiency in mice exacerbated the cytokine storm during endotoxemia and caecal ligation and puncture (10); this is associated with impaired macrophage number in the peritoneum. Complex alterations in the inflammatory response in these mice limited the use of rCLEC-2-Fc treatment during endotoxemia, due to a drastic early increase in the clinical severity. Therefore, the mechanisms driving the dysregulated inflammatory response in CLEC-2-deficient mice require further investigation.

In conclusion, we show a novel, key immunomodulatory role for CLEC-2-podoplanin interaction to limit the accumulation and retention of highly inflamed macrophages in tissues, a major complication observed in many sterile thromboinflammatory diseases such as atherosclerosis and metabolic syndrome. rCLEC-2-Fc may present a novel pathway to reduce the accumulation of inflammatory macrophages, limiting tissue inflammation and subsequent progression a to chronic inflammatory state.

## Data Availability Statement

The original contributions presented in the study are included in the article/**Supplementary Material**. Further inquiries can be directed to the corresponding author.

## Ethics Statement

The animal study was reviewed and approved by UK Home office.

## Author Contributions

JB designed and performed research, collected, analysed and interpreted data, and wrote the manuscript. NB-C performed experiments and contributed to data analysis. MZ, JC, EG, and MC performed experiments. YD and ST provided reagents. LT contributed to data analysis. AB and JB contributed to data interpretation. SW contributed to data interpretation and provided reagents. JR designed and performed research, interpreted data and wrote the manuscript. All authors contributed to the article and approved the submitted version.

## Funding

This work was supported by a College-funded PhD studentship (University of Birmingham), a BHF Accelerator Award (AA/18/2/34218) and the Centre of Membrane Proteins and Receptors. JR is supported by a BHF Intermediate Basic Science Fellowship Application (FS/IBSRF/20/25039) and Wellcome Trust 4-year studentship for MC (204951). AB is supported by BHF Senior basic Science Research Fellowship (FS/19/30/34173). SPW holds a BHF Chair (CH/03/003).

## Conflict of Interest

The authors declare that the research was conducted in the absence of any commercial or financial relationships that could be construed as a potential conflict of interest.
