# Supplementary material for: CLEC-2 Prevents Accumulation and Retention of Inflammatory Macrophages During Murine Peritonitis
Source: Front Immunol. 2021 Jun 7;12:693974. doi: 10.3389/fimmu.2021.693974 (PMC8215360; doi:10.3389/fimmu.2021.693974)
Supplement: Supplementary file 1 [file DataSheet_1.pdf]

Supplemental Methods

Recombinant protein generation

rCLEC-2-Fc was designed by cloning the ECD region of mouse CLEC2 into mammalian expression vector pFuse-rIgG-Fc (Invitrogen). The construct was used to stable transfect HEK-293T cells with PEI method. The stable line is established by Zeocin selection and function screening. rCLEC2-fc was then purified from the cell culture supernatant by protein-A affinity chromatography, followed validations by SDS-PAGE, Western Blots and various functional assays.

Protein quantification

BMDM and RAW264.7 cells were cultured with LPS (*Escherichia Coli* 055: B5) 1µg/ml for 24h. Macrophages were scraped in ice-cold PBS-EDTA (5mM), Fc-receptors blocked (anti-CD16/CD32) in 10% mouse serum, and receptor surface expression assessed by Accuri C6 Plus flow cytometry (BD Bioscience, Oxford, UK). Cytokines and chemokines were measured in the cell media supernatant by ELISA (Peprotech). For western blotting, cells were lysed in NP40 buffer supplemented with 1%PMSF, Na_3VO_4, NaF and protease inhibitor cocktail (Roche). Protein concentration was estimated by Bradford reagent (Biorad). Protein electrophoresis was performed in Tris-Glycine SDS running buffer (Biorad). Immunoprecipitation was performed in whole cell lysate using anti-podoplanin antibody (clone 8.1.1) bound to protein A-sepharose beads.

Confocal microscopy

BMDM and RAW264.7 cells were cultured with LPS (1µg/ml) for 24h before being allowed to interact with platelets in suspension for 15min. For immunofluorescence, cells were fixed in 4% paraformaldehyde (PFA), blocked (1%BSA, 5% goat serum) and stained using for nuclei using Hoechst 33342 and specific antibodies. Cells were flat-mounted using Hydromount (National Diagnostics). Images were acquired using an inverted confocal microscope (Zeiss LSM 780) using a 1.49 NA 64X oil-immersion objective. Argon-ion lasers 405nm, 457-514nm and 561nm were used to excite constructs. Cells were quantified for size and circularity using ImageJ.

LightSheet diSPIM microscopy

LPS-activated BMDM derived from LifeAct-GFP mice were allowed to spread on collagen-coated slides. Platelets from WT mice were stained with CellMask Deep Red plasma membrane stain (Invitrogen) and were allowed to interact with the BMDM for 15 min prior to imaging. Cells were imaged in phenol red free DMEM supplemented with 2% FBS at 37°C and 5% CO_2. Fluorescence was acquired on a Marianas LightSheet (Intelligent Imaging Innovations, Denver, CO, USA), a dual inverted Selective Plane Illumination Microscope (diSPIM) fitted with two perpendicular 0.8 NA, 40x water immersion objectives and ORCA-Flash4.0 V3 sCMOS cameras (Hamamatsu), driven by SlideBook software (Intelligent Imaging Innovations, Denver, CO, USA). Volumes were captured every minute in slice scan mode, with a step size of 0.5μm and 488nm and 640nm excitation wavelengths. Time lapse movies of maximal intensity projections were visualised using Arivis.

3D-Structured illumination microscopy (SIM)

BMDM were allowed to spread on collagen-coated #1.5H glass coverslips for 2h. Cells were then fixed in prewarmed 4% PFA in PEM buffer, permeabilised and blocked before immunofluorescent staining (ERM-AF488; podoplanin-AF568) and flat-mounting with VECTASHIELD antifade mounting medium (Vector Labs). SIM imaging was performed on a Nikon N-SIM-S system with Nikon Perfect Focus in 3-D SIM mode, using a Nikon 1.49x Na oil Objective, Chroma ET525/50m and ET595/50m excitation filters, and an ORCA Flash4.0 sCMOS camera (Hamamatsu). Illumination was with 488nm and 561nm lasers. Capture and subsequent reconstruction was performed in Nikon NIS Elements 5 – stack and a maximum score of less than 8 were discarded. Composite Images were visualised and adjusted for figures using ImageJ.

Scanning electron microscopy

LPS-activated BMDM were allowed to spread on collagen-coated #1.5H glass coverslips for 1h in the presence or absence of WT platelets. Cells were PBS washed, before fixation in 2.5% glutaraldehyde. Cells were treated with osmium tetroxide, and imaged using a Philips XL30 FEG ESEM in high vacuum mode at 20kV and 2500x magnification.

Phagocytosis

LPS-treated BMDM were incubated in the absence or presence of WT or CLEC-2-deficient platelets (100 platelets: 1 Mϕ) for 1h. pH sensitive, AlexaFluor488-conjugated, *Escherichia Coli* covered (K-12 strain) BioParticles ($3x10^6$ beads/condition) were added for an additional 4h at 37°C. Total green fluorescence was quantified by a mask generated by Incucyte Systems on images taken using an Incucyte SX-5 Live-Cell microscope (Satorius).

Wound healing quantification

Confluent, LPS-treated BMDM grown in DMEM were washed and scratched using a 96-pin 800µm-wide mechanical WoundMaker [30]. They were then co-cultured in the absence or presence of WT or CLEC-2-deficient platelets (100:1) or rCLEC-2-Fc (10µg/ml) for 96h at 37°C. Cells were imaged every 2h using an Incucyte SX-5 Live-Cell microscope (Satorius). Wound closure was quantified using ImageJ, and calculated as percentage of closure at each timepoint by:

$$100 - \left(\frac{wound\ size}{wound\ size\ t = 0}\right)$$

Boyden Chamber transwell assay

For BMDM chemoattractant stimulated migration, LPS-treated BMDM were incubated for 1h with rCLEC-2-Fc (10µg/ml) or IgG isotype (10µg/ml). Cells were scraped and seeded onto the upper layer of an 8µm pore polycarbonate inserts transwell (Corning). Migration of BMDM towards CCL21 (30ng/ml) was assessed after 4h at 37°C. Cells were fixed, stained with Hoechst 33342 and migrated cell nuclei counted.

Supplemental Movie 1: Inflammatory BMDM remain sessile
BMDM from LifeAct-GFP mice were incubated with LPS (1µg/ml) for 24h (Mϕ). BMDM (green) images were acquired for 1h by LightSheet diSPIM microscopy every minute. Movies are representative of 3 independent experiments.

Supplemental Movie 2: Platelets induce actin rearrangement in inflammatory BMDM
BMDM from LifeAct-GFP mice were incubated with LPS (1µg/ml) for 24h (Mϕ). WT platelet (red) and BMDM (green) images were acquired for 1h by LightSheet diSPIM microscopy every minute. Movies are representative of 3 independent experiments.

Supplemental Figure 1

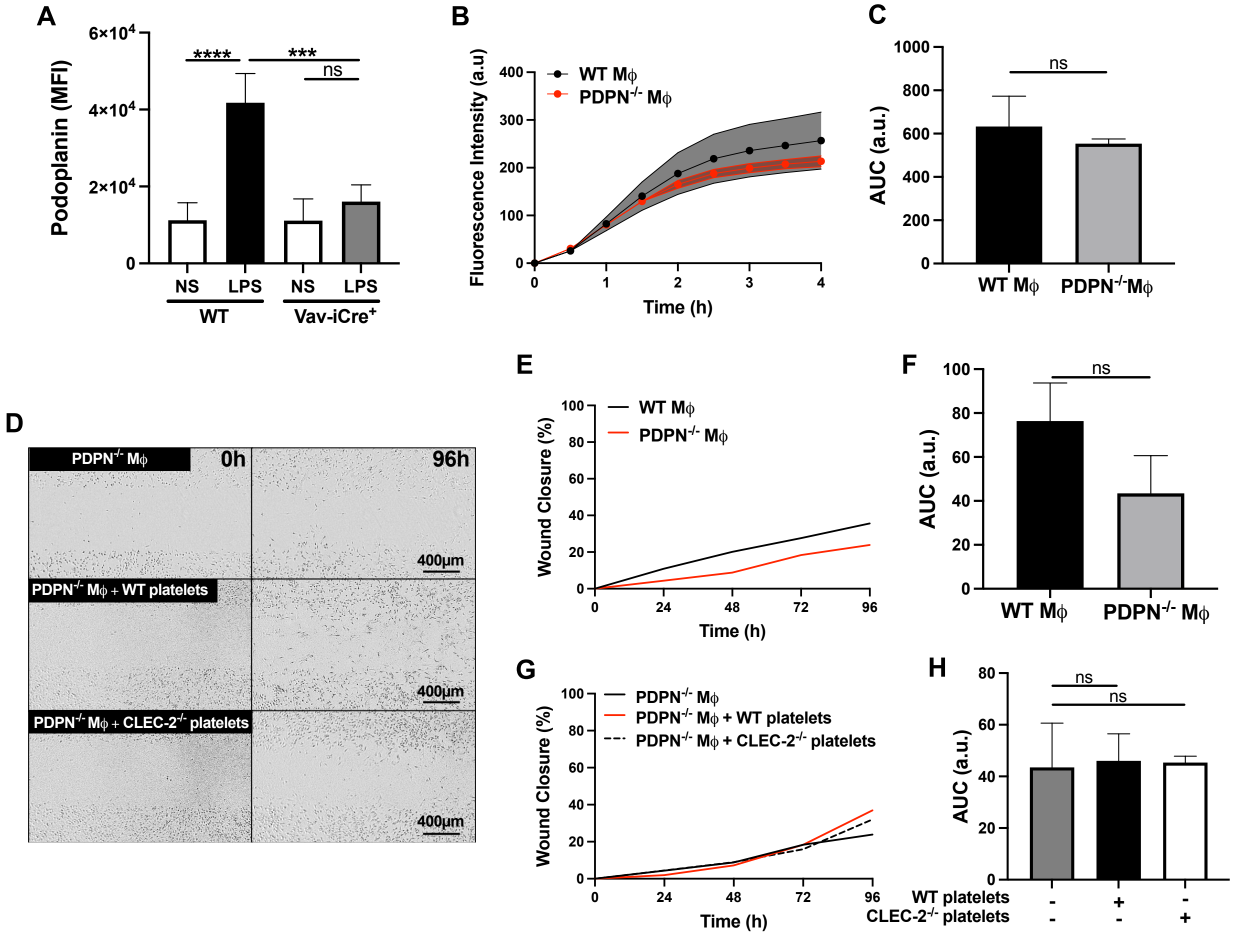

Supplemental Figure 1: Podoplanin deficiency does not reduce BMDM phagocytic capacity, but inhibits platelet-induced wound closure

(A-H) Wild type (WT) or PDPN$^{fl/fl}$Vav-iCre+ BMDMs were unstimulated (NS), or LPS-stimulated (Mϕ; 1µg/ml) for 24h. **(A)** Mean fluoresence of intensity (MFI) of podoplanin was detected by flow cytometry (n=4). **(B, C)** Phagocytosis of pH sensitive Alexa Fluor-488 conjugated *Escherichia Coli* bioparticles (3x10^{6} beads/condition) by WT or podoplanin-deficient (PDPN$^{-/-}$) macrophages was visualized for 4h and analyzed using Incucyte SX-5 Live-Cell microscopy. (n=4). **(D-H)** Scratch wound migration of PDPN$^{-/-}$Mϕ was monitored every 2h for 96h using an Incucyte ZOOM system. Following wound scratch, **(G)** WT or CLEC-2-deficient platelets (100 platelets:1 M) were added to Mϕ. **(E, G)** Wound closure was quantified as percentage of closure using ImageJ. **(F,H)** Total wound closure was quantified by AUC at 96h (n=3). The statistical significance between 2 groups was analyzed using a student's paired t-test and the statistical difference between multiple groups using one-way ANOVA with Tukey's multiple comparisons test. ***$p < 0.001$, **** $p<0.0001$.

Supplemental Figure 2

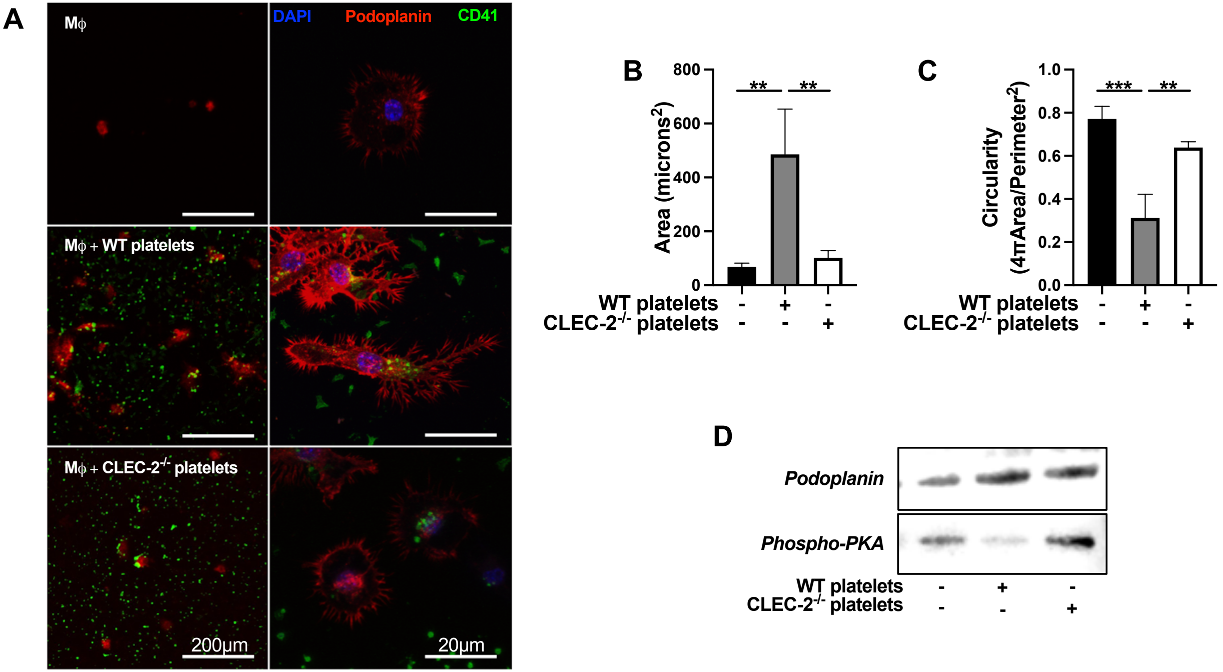

Supplemental Figure 2: Platelet CLEC-2 induces spreading and filopodia formation

(A) LPS-activated (1μg/ml) RAW 264.7 cells were cultured on glass, and WT- or CLEC-2-deficient platelets added for 1h (100:1). Cells were fixed, permeabilized and nuclei (Hoechst 33342, blue), podoplanin (red) and platelets (CD41, green) were detected using confocal microscopy. **(B)** Cell area and **(C)** circularity were analyzed using ImageJ (n=3). **(D)** Podoplanin was immunoprecipitated with anti-podoplanin antibody (8.1.1), and immunostained with anti-phospho-PKA substrate antibody. Representative western blot of 3 independent experiments. The statistical significance between 2 groups was analyzed using a student's paired t-test and the statistical difference between multiple groups using one-way ANOVA with Tukey's multiple comparisons test. *$p < 0.05$ **$p < 0.01$ ***$p < 0.001$.

Supplemental Figure 3

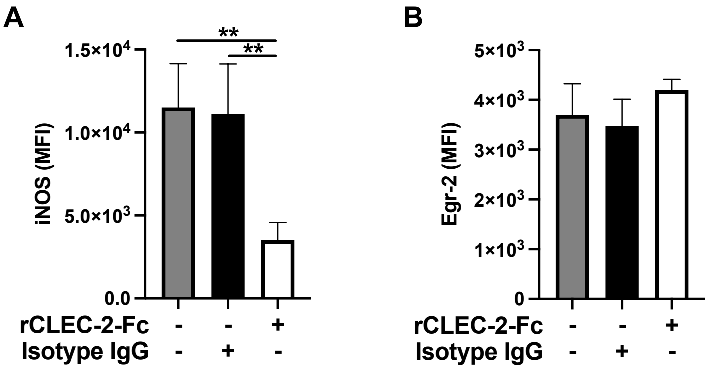

Supplemental Figure 3: rCLEC-2-Fc reduces M1 phenotype, but does not modify polarization

(A-B) LPS-activated BMDM (1μg/ml) were cultured with rCLEC-2 (10μg/ml) or IgG istopye control (10μg/ml). Median of fluoresence intensity (MFI) for (**A)** iNOS and **(B)** Egr-2 was detected by flow cytometry (n=4). The statistical difference between multiple groups using one-way ANOVA with Tukey's multiple comparisons test. $^{**}p < 0.01$

Supplemental Figure 4

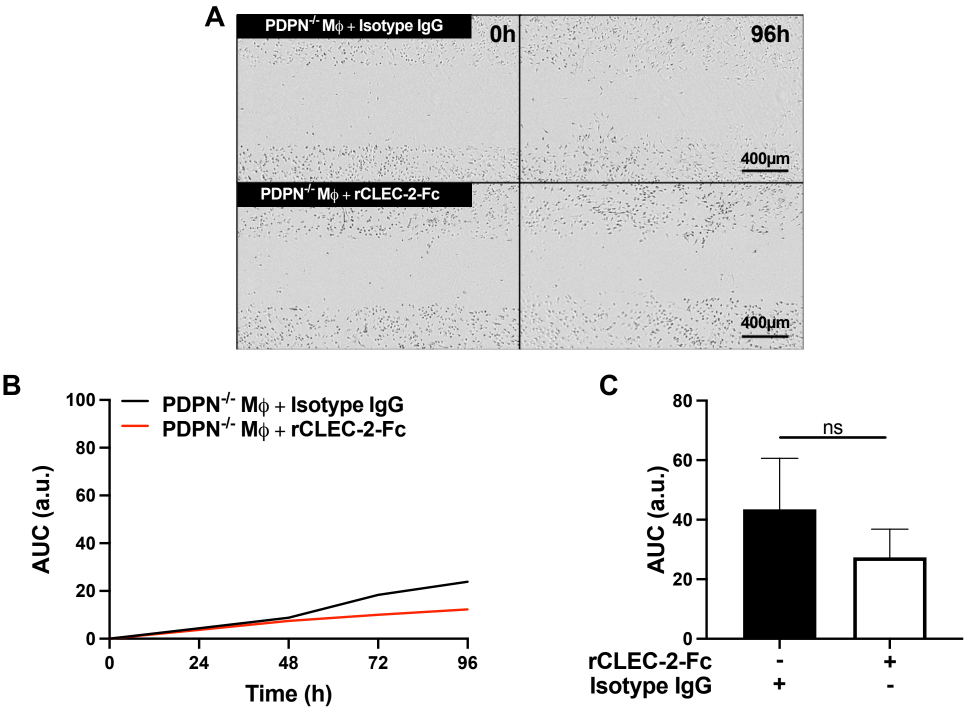

Supplemental Figure 4: BMDM podoplanin deficiency inhibits rCLEC-2-FC induced wound closure

(A-C) PDPN$^{fl/fl}$Vav-iCre+ BMDMs were LPS-stimulated (Mϕ; 1μg/ml) for 24h. Scratch wound migration of PDPN$^{-/-}$Mϕ was monitored every 2h for 96h using an Incucyte ZOOM system. Following wound scratch, **(A)** Isotype IgG (10μg/ml), or rCLEC-2-Fc was added to Mϕ (10μg/ml). **(B)** Wound closure was quantified as percentage of closure using ImageJ. **(C)** Total wound closure was quantified by AUC at 96h (n=3). The statistical significance between 2 groups was analyzed using a student's paired t-test.

Supplemental Figure 5

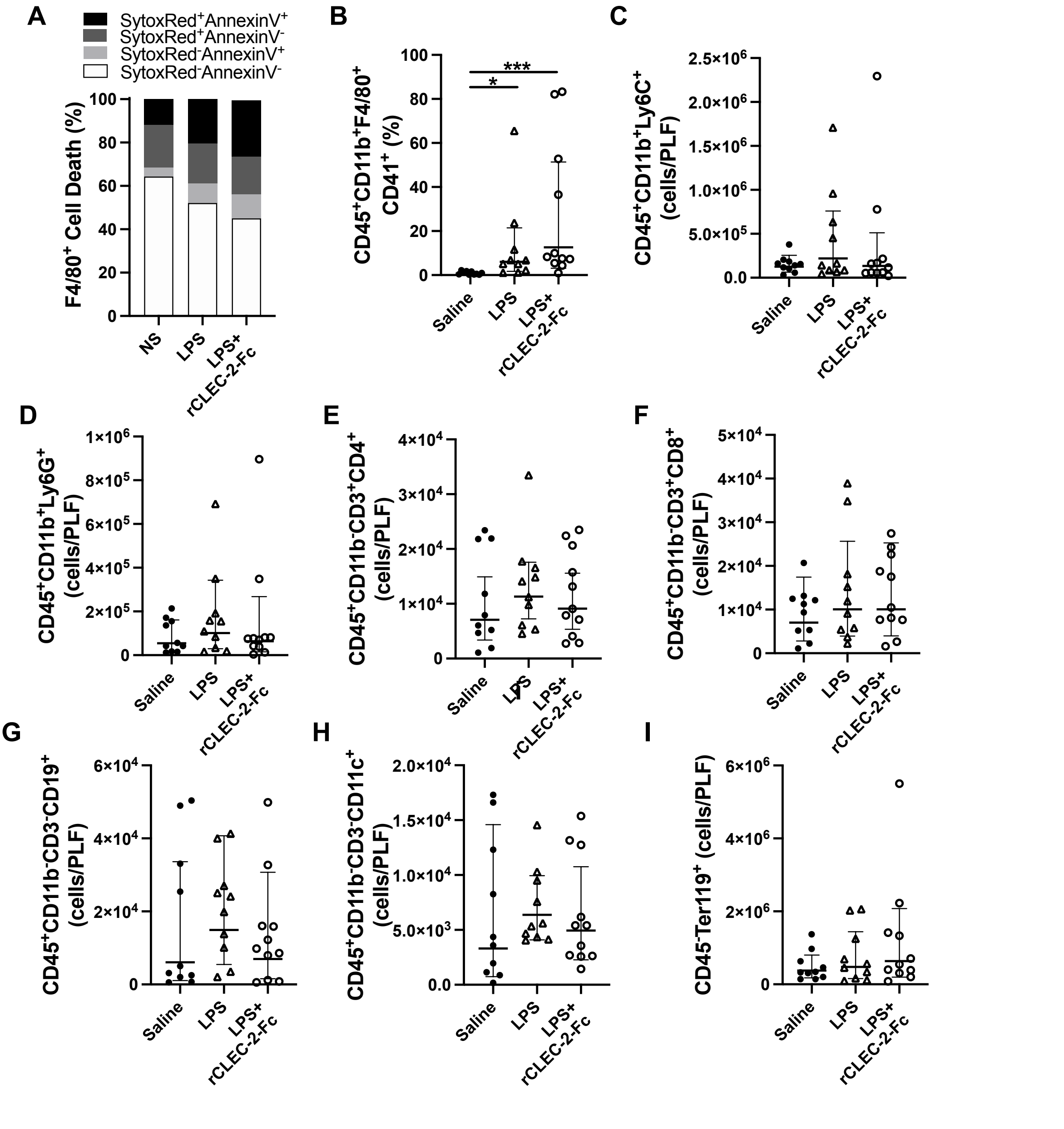

Supplemental Figure 5: rCLEC-2-Fc does not significantly alter macrophage death or platelet-macrophage complexes in the inflamed peritoneum

WT mice were intraperitoneally injected with LPS (10mg/kg) for 18h followed by rCLEC-2-Fc or IgG isotype control (100µg/mouse) for and additional 4h (n=11). **(A)** Peritoneal macrophage ($CD45^{+}CD11b^{+}F4/80^{+}$) viability was quantified using SytoxRed and AnnexinV staining. **(B)** The percentage of platelet-macrophage complexes in the peritoneum ($CD45^{+}CD11b^{+}F4/80^{+}CD41^{+}$), total count of **(C)** monocytes ($CD45^{+}CD11b^{+}Ly6C^{+}$) and **(D)** neutrophils ($CD45^{+}CD11b^{+}Ly6G^{+}$) in the peritoneum were measured by flow cytometry. Total count of **(E)** CD4+ and **(F)** CD8+ T cells ($CD45^{+}CD11b^{-}CD3^{+}CD8^{+}$), **(G)** B cells ($CD45^{+}CD11b^{-}CD3^{-}CD19^{+}$), **(H)** dendritic cells ($CD45^{+}CD11b^{-}CD3^{-}CD19^{-}CD11c^{+}$) and **(I)** erythrocytes ($CD45^{-}Ter119^{+}$). The statistical significance was analyzed using a Kruskal-Wallis test. $^{*}p < 0.05$ $^{***}p < 0.001$.

Supplemental Figure 6

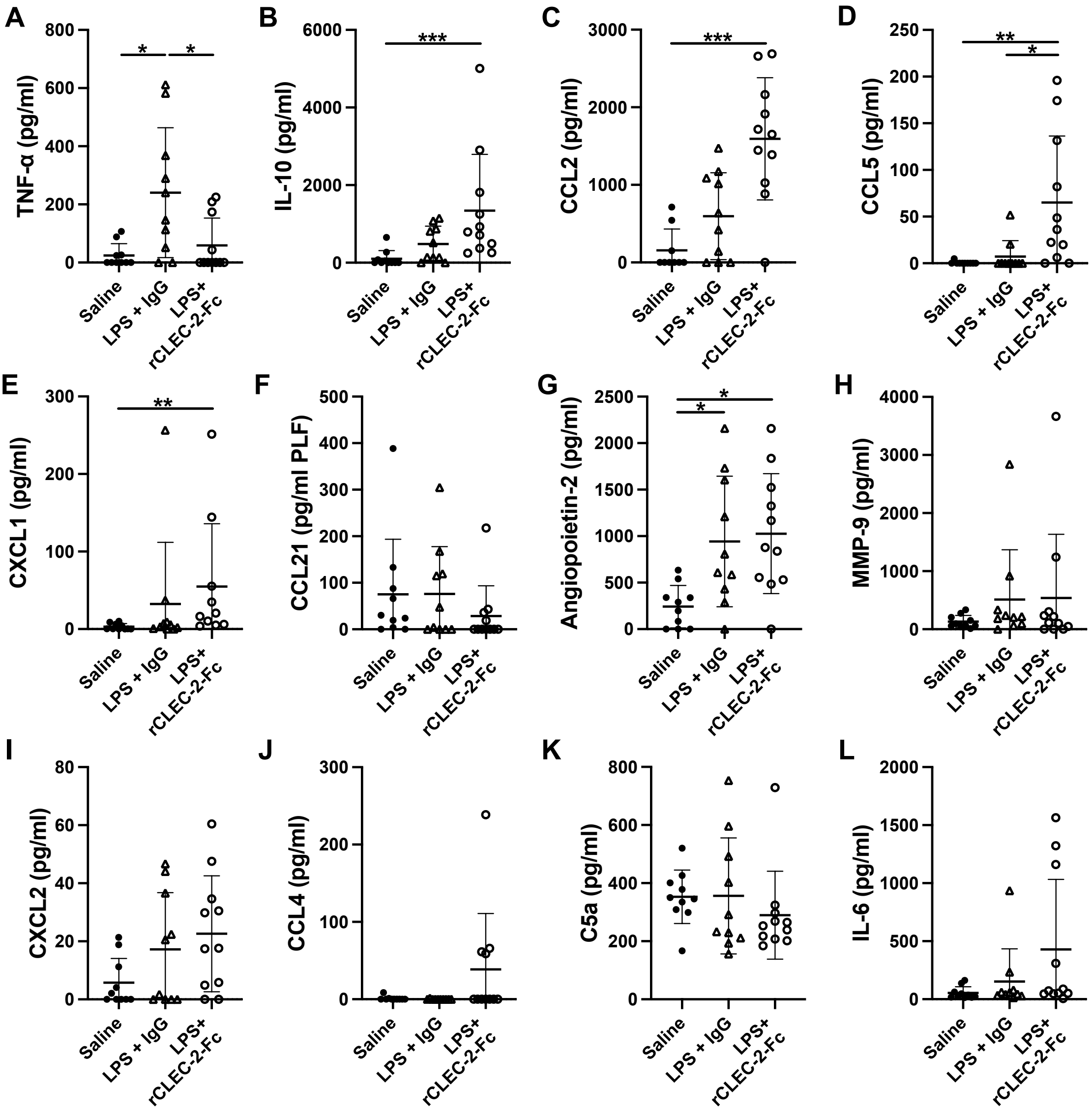

Supplemental Figure 6: Peritoneal cytokine and chemokine profile during endotoxemia

WT mice were intraperitoneally injected with LPS (10mg/kg) for 18h followed by rCLEC-2-Fc or IgG isotype control (100µg/mouse) for and additional 4h (n=11). **(A-L)** Chemokine and cytokine secretion in the peritoneal lavage fluid (PLF) of **(A)** TNF-α, **(B)** IL-10, **(C)** CCL2, **(D)** CCL5, **(E)** CXCL1, **(F)** CCL21 **(G)** angiopoietin-2, **(H)** MMP-9, **(I)** CXCL2, **(J)** CCL4, **(K)** C5a, and **(L)** IL-6 was measured by ELISA. The statistical significance was analyzed using a Kruskal-Wallis multiple comparisons test. $^{*}p < 0.05$ $^{**}p < 0.01$ $^{***}p < 0.001$

Supplementary Table 1

Antibody (clone)	Host/Conjugate	Reference	Source
Podoplanin (8.1.1)	PE	127408	BioLegend
Podoplanin (8.1.1)	Syrian Hamster	14-5381-85	eBioScience
CD44 (IM7)	AF647	103018	BioLegend
CD44 (IM7)	Rat	70-0441-U100	Tonbo Bioscience
CD41 (MWReg30)	PE	12-0411-82	invitrogen
CD41 (MWReg30)	BV421	133912	BioLegend
CD41 (MWReg30)	FITC	133904	BioLegend
CD41 (MWReg30)	Rat	553847	BD Bioscience
ERM	Rabbit	3142S	Cell Signalling
CD45 (30-F11)	APC	20-0451-U100	Tonbo Bioscience
CD45 (30-F11)	APC-Cy7	47-0451-82	Invitrogen
CD11b (m1/70)	Biotin	13-0112-82	eBioScience
F4/80 (BM8)	eFluor450	48-4801-82	eBioScience
F4/80 (BM8)	Rabbit	ab100790	abcam
F4/80 (BM8)	APC	17-4801-82	eBioScience
CLEC-2	FITC	-	internally sourced
Ly6C (HK1.4)	Rat	128002	Biolegend
Ly6C (HK1.4)	PERCP-Cy5.5	45-5932-82	eBioScience
Ly6G (1A8)	V450	560603	BD Horizon
CD80 (16-10A1)	FITC	35-0801-U500	Tonbo Bioscience
β-Actin (polyclonal)	Rabbit	ab8227	abcam
Phospo-PKA (RRXS*/T*	Rabbit	9624S	Cell Signalling
AnnexinV	FITC	556420	BD Pharmingen
SytoxRed	TxRed	S34859	ThermoFisher
CD3 (145-2C11)	PE-Cy7	25-0031-82	eBioScience
CD4 (RM4-5)	PE-Cy5.5	35-0042-82	eBioScience
CD8a (53-6.7)	PE	12-0081-82	eBioScience
CD11c (N418)	FITC	11-0114-82	eBioScience
Ter119 (TER119)	Rat	116202	Biolegend
CD16/32 (93)	Rat	14-0161-86	eBioScience
CD19 (eBio1D3)	APC-Cy7	47-0193-82	eBioScience
LYVE1 (polyclonal)	Rabbit	ab14917	abcam
CCL21 (polyclonal)	Biotinylated	BAF457	R&D

Supplemental Table 1: Antibodies used for immunofluoresent imaging.
